# Assessment of Convergence Angle of Tooth Preparations for Complete Crowns Among Dental Students: Typodont vs Simulator

**DOI:** 10.1155/2022/7615892

**Published:** 2022-05-10

**Authors:** Meriem Amine, Hafsa Oumayma Wahid, Saliha Fahi, Safaa Lehmouddi, Mouna Hamza, Samira Elarabi

**Affiliations:** Faculty of Dentistry of Casablanca, Hassan II University of Casablanca, Casablanca, Morocco

## Abstract

**Introduction:**

The technology-enhanced learning and simulation-based learning are critically important pedagogic tools. They allow students to perfect their preclinical training by improving their skills and their manual dexterity while facilitating the acquisition of the know-how necessary for reproduction more realistically and faithfully of the behaviors required for a better dental practice. Retention is one of the mechanical fundamental principles of preparation of cemented fixed prostheses. It depends on several factors including the convergence of the axial walls. The undercut must be sparing in the reduction of tissue volume to obtain a low degree of convergence and a sufficient height of the preparation to comply with the retention and stabilization requirements of the prosthetic element. A draft value of 6° was recommended initially, but a range extending up to 16° has been accepted according to Weed et al. and Dodge et al., as being clinically achievable while providing good retention. Are students able to reproduce, in preclinical, total occlusal convergence (TOC) angles recommended on typodont and simulator?

**Objective:**

The evaluation of the TOC of the preparations made on typodont and simulator by the students in the 3^rd^ year of the Faculty of Dental Medicine of Casablanca (FDMC). *Material and Methods*. A total of 140 dental preparations for cast crowns and metal-ceramics made by thirty-five 3^rd^ year FDMC students were scanned by using the IDENTICA HYBRID optical scanner. The STL files were read by the 3D-TOOL-FREE software, two images were extracted for each preparation using the screen capture tool, and the two mediodistal (MD) and buccolingual angles (BL) were measured by the MB-RULER software. The statistical data were analyzed using the SPSS, software and comparisons were made by Student's *t*-test.

**Results:**

An overall average of 11.99° ± 4.48 was recorded for the preparations on the typodont with 11.40° ± 5.09 in the MD direction and 12.58° ± 4.74 in the BL direction. Concerning the simulator preparations, we recorded an overall average of 11.31° ± 4.16 with 10.81° ± 4.29 in the MD direction and 11.80° ± 5.44 in the BL direction. No significant difference was observed when comparing the preparations made on the typodont and the simulator. A percentage of 68.6% and 74.3% of the preparations made on the typodont and the simulators respectively fall within an acceptable range of 6 to 16°. *Discussion*. The TOC values achieved by the majority of students correspond to the recommended values which are 6 to 16° on average. The results of the simulator preparations are similar to the results of Marghalani for dental students at King Abdulaziz University, and Tiu et al., at the University of Otago in New Zealand.

**Conclusion:**

This study highlighted the difference between what is taught in dental schools, which is theoretically possible, and the academic results of actual practice. The generally recommended 6° tapers have proven difficult to achieve for many dental students. A margin of 6° to 16° of TOC angle is clinically achievable and allows sufficient retention. We can retain that most of the sample of our study had values that fall within the recommended range.

## 1. Introduction

The principles of tooth preparation play a key role in the success of prosthetic rehabilitations and are known to influence retention and strength of the latter, so it is a key skill in restorative dentistry, and teaching this procedure is an important part of predoctoral dental study programs.

The retention and strength of cement-retained crowns depend on various factors such as the convergence angle of the preparation, the height of the preparation, the sealing cement, and the preparation surface.

The convergence angle of tooth preparation is the combined angle formed by opposing axial walls to the long axis of the tooth.

Theoretically, the more parallel the tooth preparation opposing walls are, the greater is the retention. It is widely accepted that the convergence angle of a crown preparation should be as close to parallel as possible to achieve adequate retention and strength. However, the insertion of the prosthetic element requires the arrangement of a draft represented by a convergence of the axial walls towards the occlusal face of an ideal value of 6° recommended academically but faced with the difficulty of to be so precise in preparation, a clearance margin of 6° to 16° is accepted by Weed et al. and Dodge et al., as being clinically achievable while still providing good retention [1–4].

Practical preclinical teaching aims to enable students to build skills as the professional situations they encounter emerge.

So are the students able to reproduce on typodonts and on simulators the values of angles of total occlusal convergence (TOC) recommended?

The objective of this study is to measure and evaluate the TOC of dental preparations made on typodonts as well as on simulators by 3^rd^-year FDMC students.

## 2. Materials and Methods

Thirty-five 3^rd^-year FDMC students, randomly drawn were included in this study.

Each student made two preparations on the typodont, one for a metal-ceramic crown MCC on the incisor and the second for a cast crown CC on the molar. Then, the same preparations were made on a simulator, in the working position.

One hundred and forty dental preparations were made, and they were scanned by using the IDENTICA HYBRID scanner, yielding 70 STL files.

Using the 3D-TOOL-FREE software and screen capture tool, four images were extracted from each STL file.

Each preparation gives two images: one with a buccolingual (BL) view and the other with a mediodistal (MD) view.

Using the Paint tool, lines were drawn parallel to the axial walls of the buccal, lingual, medial, and distal surfaces forming a TOC angle of each face.

The image processing was carried out using the MB-RULER software, making it possible to measure the angles of convergence using the graduated square.

The data analysis consisted of a descriptive analysis including calculations of percentages and averages and then, a comparative analysis using the *t*-test.

## 3. Results

A total of 140 preparations including 70 on typodont and 70 on simulator were analyzed.

35 students including 22 females (62.9%) and 13 males (35.1%) participated in the study.

An average overall TOC of 11.99° ± 4.48 was recorded for the preparations on the typodont with 11.40° ± 5.09 in the MD direction and 12.58° ± 4.74 in the BL direction. However, an overall average of 11.31° ± 4.16 with 10.81° ± 4.29 in the MD direction and 11.80° ± 5.44 in the BL direction was recorded on the simulator preparations. No significant difference was observed when comparing the preparations made on the typodont and the simulator (*p*=0.294) (Table 1).

The mean overall TOC of typodont preparations performed by female students was 11.50°± 4.08 and 12.80°± 5.14 for male students. The difference in results by gender for typodont preparations was not significant (*p*=0.413).

The mean Overall TOC of the simulator preparations performed by the female students was 10.45°± 3.57 and 12.75°± 4.79 for the male students. The difference in results by gender for the simulator preparations was not significant (*p*=0.113).

A percentage of 68.6%, of which 45.7% were female and 22.9% were male passed preparations on typodonts with a TOC within the range (6°–16°).

A percentage of 74.3%, of which 48.6% were female and 25.7% were male passed simulator preparations in the same range (Table 2).

The average Overall TOC of the incisors was 9.27 ± 4.64, that of the molars was 14.70 ± 5.23 for the typodont preparations. For simulator preparations, an average TOC of 9.13 ± 3 .73 was recorded for incisors and 13.48 ± 5.46 for molars. The difference in the results according to the type of teeth was significant for both the preparations on the typodont (*p*=0.000018) and on the simulator (*p*=0.000218) (Table 3).

The mean overall TOC of the MCC preparations was 9.27 ± 4.64 on the typodont and 9.13 ± 3.73 on the simulator. The mean overall TOC of the CC preparations was 14.70 degrees ± 5.23 on the typodont and 13.48° ± 5.46 on the simulator. The difference in results according to the type of preparations for those on the typodont and the simulator was significant (respectively *p*=0.000018 and *p*=0.000218) (Table 4).

## 4. Discussion

The clinical success of crown retention depends on several factors. The taper, height, volume of remaining tooth structure, and type of cement all affect the success of the final restoration. Researchers disagree on the ideal taper, which requires manual dexterity that is difficult to achieve, even for experienced clinicians [5].

Prosthodontic manuals recommend an optimal convergence angle value of 6° [3] and according to Shillingburg, this angle would correspond to the 3° inclination given by the conical cutters to each axial surface. When this convergence is less than 6°, there is an undercut, which leads to an insertion problem, or on the contrary, compromised retention when this angle is greater than 16°. For Jørgensen, there is an inverse relationship between convergence angle and retention (in6). However, this theoretical recommendation is difficult to achieve in clinical practice.

Several clinical studies have been carried out with the aim to establish optimal convergence angles in clinical practice and providing acceptable long-term retention. A convergence of 16° is considered the most clinically acceptable, and it provides satisfactory retention [2–4].

In our study, this value exceeds 6° but falls within the recommended range of (6°–16°) for a percentage of 68.6% of students. Similar results were published in the multicenter study by Ayad et al. [6], which showed a clearance value of 19.8°± 10 in the BL sense and 19.4 ± 9.1 in the MD sense for Tanta University students, 17.3 ± 5.9 in the BL sense and 15.2°± 4.6 in the MD sense for Ohio University students and a value of 15, 6 ± 4.8 in the BL direction and 14.1 ± 3.8 in the MD direction for King Abdulaziz University students.

For an interval of (4°–12°) recommended in the same study [6], 23% of the preparations of Egyptian students, 35% of those of United States students, and 47% for Saudi students had angles of convergence deemed acceptable.

Regarding simulator preparations, the recommended interval is respected for 74.3% of our students. These values are similar to those recorded in the study of Marghalani at King Abdulaziz University; mean convergence angles were between 10.16° and 11.46°, with 11.11 ± 4.79 in MD and 10.49°± 3.90 in BL [7].

The results of the preparations on typodonts showed that 45.7% of the female sex and 22.9% of the male sex respected the recommended interval (6°–16°) and for the preparations on the simulator, the percentages were 48.6% for the female sex and 25.7% for the male sex. These results are also confirmed by Marghalani [7].

Mobaraki et al. reported a significant difference between the male and female sex concerning MD reduction [8].

Regarding the values of the TOC according to the type of teeth, the results of our study are similar to those of Kirov et al. [9] who obtained an overall TOC value of 13.92° for the molars which means that it falls within the range of recommended values.

Similarly, Yoon et al. [10] found TOC values for preparations made on simulators of 6.3° in MD and 20.4° in BL for incisors and molars of 16.9° in MD and 16.3° in BL and thereafter these averages respected the recommended values except in BL of the incisors.

The preparations for CC were performed on the first molar and MCC on centrale incisor.

Strain et al. found that dental students could develop dental preparations for full crowns with a taper of 12.78 on typodonts [5].

Noonan and Goldfogel [11] studied 909 complete dental crown preparations prepared by students for gold crowns and reported an overall average taper of 19.28°. Nordlander et al. analyzed 208 dental preparations by 10 dentists and reported a minimum of 17.38° for premolars and a maximum of 27.38° for molars, with an overall mean of 19.98°.

The integration of virtual reality simulators allows students to watch and evaluate their preparations on a monitor [12]. Haptic technology offers an additional dimension to virtual reality thanks to the sense of touch and the force feedback of the different dental and bone structures.

Thus, it is effective in training dental students in hand-eye coordination and spatial reasoning skills. It has also helped students improve their preparations with precision, shorten preparation time in the very early stages of training, and increase a conservative preparation approach [13].

The complexity of prosthetic procedures requires significant precision, and therefore the concept of creating digital clinical scenarios is extremely beneficial. Experimental trials using virtual reality simulators for crown and bridge exercises have demonstrated significant improvement in student scores in various clinical applications of prosthodontics. The introduced virtual reality simulators allowed students to keep track of their progress while simultaneously viewing their cases, thus offering promising results during the training process [12].

As in any methodical research, several difficulties were encountered during this study, the most important of which are the incompatibility of several types of scanners with FRASACO teeth and the difficulties encountered when downloading and handling software.

## 5. Conclusion

The optimal convergence angle for maximum retention is approximately 6°, meaning that the axial walls of the restoration are angled at 3° relative to the long axis of the tooth. However, in a clinical position, it is difficult to make a similar preparation.

It is therefore accepted that an interval of 6° to 16° is easily achievable clinically and allows sufficient retention.

An acceptable percentage of students succeeded in obtaining convergence angles following the recommendations of the literature.

The diversity of student learning styles and motivation is a crucial challenge facing course designers. The introduction of virtual simulators into the dental curriculum to predict clinical performance provides the opportunity to tailor the learning process to meet individual student diversity and allow students to work at their own pace. In this context, the dental curriculum could provide an education that leads to the optimal performance of each student.

## Figures and Tables

**Table 1:** Average TOC of typodont and simulator preparations.

TOC values	On typodont (degree)			On simulator (degree)		
	MD	BL	Overall average	MD	BL	Overall average
Mean	11.40	12.58	11.99	10.81	11.81	11.31
Standard deviation	5.09	4.74	4.48	4.29	5.44	4.16

**Table 2:** Distribution of students according to TOC values on typodont and simulator by sex.

TOC values	On typodont		On simulator	
	Women %	Men %	Women %	Men %
<6°	5.7	2.9	8.6	2.9
6°–16°	45.7	22.9	48.6	25.7
>16°	11.4	11.4	5.7	8.6

**Table 3:** Average TOC on typodont and simulator according to the type of teeth.

	On typodont		On simulator	
	Incisors	Molars	Incisors	Molars
MD mean (standard deviation)	7.05 ± (5,82)	15.74 ± (5.97)	6.88 ± (4.73)	14,74 ± (5.63)
BL mean (standard deviation)	11.49 ± (5,12)	13.67 ± (6.17)	11.38 ± (5.22)	12,23 ± (6.85)
Global mean (standard deviation)	9.27 ± (4,64)	14.7 ± (5.23)	9.13 ± (3.73)	13,48 ± (5.46)

**Table 4:** Average TOC on typodont and simulator according to the type of preparations.

	On typodont		On simulator	
	Ceramic-metal crown	Cast crown	Ceramic-metal crown	Cast crown
MD mean (standard deviation)	7.05 ± (5.82)	15.74 ± (5.97)	6.88 ± (4,73)	14.74 ± (5.63)
BL mean (standard deviation)	11.49 ± (5.12)	13.67 ± (6.17)	11.38 ± (5.22)	12.23 ± (6.85)
Global mean (standard deviation)	9.27 ± (4.64)	14.7 ± (5.23)	9.13 ± (3.73)	13.48 ± (5.46)

## Data Availability

The data used to support the conclusions are included within the paper,and more details are available from the corresponding author upon request.
